# Probiotics ameliorate atopic dermatitis by modulating the dysbiosis of the gut microbiota in dogs

**DOI:** 10.1186/s12866-025-03924-6

**Published:** 2025-04-22

**Authors:** Hyokeun Song, Seung-Hyun Mun, Dae-Woong Han, Jung-Hun Kang, Jae-Uk An, Cheol-Yong Hwang, Seongbeom Cho

**Affiliations:** 1 College of Veterinary Medicine and Research Institute for Veterinary Science, Seoul National University, Seoul, South Korea; 2 Comparative Medicine Disease Research Center, Seoul National University, Seoul, South Korea; 3 Center for Veterinary Integrated Medicine Research, Seoul National University, Seoul, South Korea

**Keywords:** Canine atopic dermatitis, Dysbiosis, Dog, Gut microbiota, Probiotics

## Abstract

**Background:**

Canine atopic dermatitis (cAD) is a chronic inflammatory disease that significantly reduces the quality of life in dogs. Dysbiosis of the gut microbiota affects skin diseases through the gut–skin axis. Therefore, microbiota-targeted therapy may potentially serve as a new management strategy for cAD. The present study aimed to investigate the association between gut microbiota and cAD and to evaluate the effect of probiotics on the clinical symptoms of cAD and gut microbiota in dogs.

**Results:**

Gut microbiota was analyzed at baseline and after 8 and 16 weeks. Baseline analysis revealed significantly lower (*p* < 0.05) gut microbial diversity in dogs with cAD than in healthy dogs. Differential abundance analysis showed that *Fusobacterium*, *Megamonas*, *Collinsella*, *unclassified Clostridiales*, *Bacillus*, *Helicobacter,* and *Caproiciproducens* were significantly more abundant in healthy dogs. In contrast, *Clostridioides*, *Erysipelatoclostridium, Clostridium, Terrisporobacter,* and *unclassified Ruminococcaceae* were significantly more abundant in dogs with cAD, In addition, differential abundance analysis showed that the abundance of 46 metabolic pathways were significantly different between healthy dogs and dogs with cAD indicating the dysbiosis of the gut microbiota in cAD. Moreover, the clinical severity of cAD was negatively correlated (*p* < 0.05) with alpha diversity and the abundance of *Fusobacterium* and *Megamonas*. Notably, daily probiotic administration for 16 weeks significantly decreased the clinical severity (*p* < 0.05). Dogs with good prognoses exhibited significantly increased alpha diversity, whereas those with poor prognoses did not, suggesting that the therapeutic effects of probiotics may be mediated by changes in gut microbial diversity.

**Conclusions:**

This study highlights the association between gut microbiota dysbiosis and cAD in dogs and demonstrates that probiotic administration can effectively ameliorate cAD by improving gut microbial dysbiosis. These findings provide a basis for novel microbiota-based therapies in cAD treatment.

**Supplementary Information:**

The online version contains supplementary material available at 10.1186/s12866-025-03924-6.

## Background

Canine atopic dermatitis (cAD) is a chronic inflammatory disease that impairs the quality of life of dogs and their owners. Potential disease causes include increased allergen load, heightened exposure to pollutants, and genetic background [1]. The association between gut microbiota and cAD is a growing research field, as the gut–skin axis is increasingly recognized in health and disease. For instance, a previous study found significant alterations in gut microbiota diversity and composition in dogs with cAD compared with those in healthy dogs, suggesting an association with the disease [2]. This preliminary study reported decreased alpha diversity of the gut microbiota in dogs with cAD, indicating dysbiosis of gut microbiota. Similarly, another study observed dysbiosis in both skin and gut microbiota of Shiba Inu dogs with cAD [3] and reported a shift in the gut microbiota, including increased alpha diversity and the abundance of Fusobacteria in dogs with cAD treated with oclacitinib. Moreover, another study of the gut and oral microbiota of dogs with cAD reported an enrichment of *Anaerovoraceae* in the gut microbiota and a reduced diversity of the oral microbiota of dogs with cAD [4]. Although these findings highlight the possible association between the gut microbiota and cAD, the specific relationship among probiotics, gut microbiota, and cAD remains unclear.

Probiotics are live microbes that are beneficial to host health when administered at suitable doses [5]. They provide health benefits to the host by enhancing gut integrity, modulating the immune system, and producing metabolites. Moreover, probiotics modulate the gut microbiota through nutrient competition with gut pathogens and the production of anti-microbial agents, including short-chain fatty acids and bacteriocin, resulting in a decrease in pathogenic bacteria in the gut [6, 7]. Therefore, probiotics are widely used owing to their potential in preventing and treating infectious, gastrointestinal, and metabolic diseases in veterinary medicine [8, 9]. Moreover, probiotics may improve feed intake and immunity in dogs [10]. In addition, probiotics are employed to mitigate the negative effects of consuming foods not recommended for dogs. For instance, probiotics promote weight loss in obese dogs with high-fat diets by altering their gut microbiome and metabolism [11]. Notably, previous clinical trials in dogs have reported that probiotic administration improves the clinical symptoms of cAD [12, 13]. These studies demonstrated that the administration of single probiotic strains, such as *Lactobacillus sakei* or *Lactobacillus rhamnosus GG*, significantly decreases the CADESI score in dogs with cAD. These findings suggest that probiotics may serve as an effective therapy for cAD. However, the specific effect of probiotics on the dynamics of the gut microbiota in cAD remains unclear.

Considering the frequent clinical use of probiotics and their demonstrated effectiveness as auxiliary treatments for cAD in dogs, we hypothesized that gut microbiota dysbiosis is associated with cAD severity and that probiotic administration ameliorates cAD severity by modulating the gut microbiota. Therefore, this study aimed to investigate: 1) the association between gut microbiota dysbiosis and cAD and 2) the therapeutic effect of probiotics on the clinical symptoms and gut microbiota of dogs with cAD.

## Materials and methods

### Study design

Eleven privately owned, clinically healthy dogs and 23 dogs with cAD were enrolled in the present study. Written consent was obtained from the owners after a thorough explanation of the study was provided. The exclusion criteria that were applied to minimize confounding factors that may have affected the gut microbiota were as follows: (i) use of antibiotics two weeks prior to the study, (ii) dogs less than nine months old, and (iii) dogs fed a weaning diet. Detailed information on participant dogs is provided in Table S1.

**Fig. 1:**
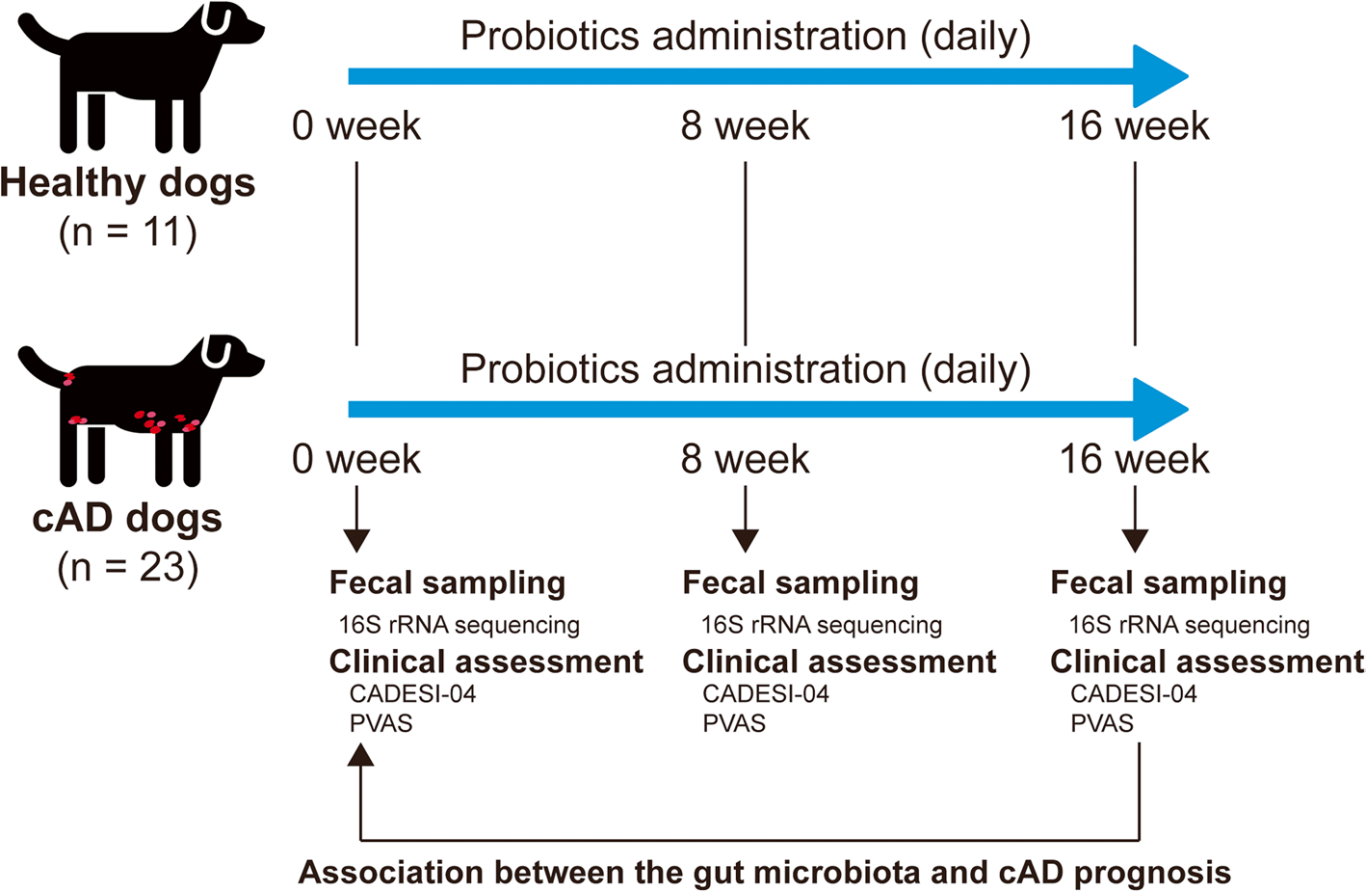
Graphical scheme of the study design. In this study, 23 dogs with canine atopic dermatitis and 11 healthy dogs were enrolled and administered probiotics for 16 weeks. Clinical assessment and microbiota analyses were performed at baseline and 8 and 16 weeks after probiotic administration

All dogs were administered commercial probiotics (Estien Co., Ltd. Gyeongsangbuk-do, South Korea) daily for 16 weeks. The probiotics comprised three bacterial strains: *Bifidobacterium bifidum*, *Lactobacillus acidophilus*, and *Enterococcus faecium*, each with a concentration of 5 × 10^7^ colony-forming units per gram. The participating dogs visited the veterinary hospital at the start of administration (baseline) and at 8 and 16 weeks after the start of administration. Each time a dog visited, veterinary dermatologists conducted physical examinations, clinical assessments, and fecal sampling. The overall study design is shown in Fig. 1.

### Clinical assessment of cAD severity

Each visit, clinical scores were measured using two indices: the fourth version of the Canine Atopic Dermatitis Extent and Severity Index (CADESI-4) and the Pruritis Visual Analog Scale (PVAS) [14]. CADESI-4 was evaluated by veterinary dermatologists, and PVAS was graded by the dog owners. Detailed information on CADESI-4 and PVAS scores of participant dogs is provided in Table S1.

### Microbiota sequencing, bioinformatics, and statistical analysis

Fecal DNA was extracted as previously described [15]. Briefly, DNA was extracted from fecal samples using a Fast DNA Soil Kit (MP Biomedicals, CA, USA), as per the manufacturer’s instructions. The 16S rRNA V3-V4 hypervariable gene region was sequenced using primers 341F and 805R (Illumina Inc., CA, USA). PicoGreen was used to pool and normalize the amplified products. All sequencing procedures were performed using the Illumina MiSeq platform at Macrogen Inc. (Seoul, South Korea).

Raw sequence data were processed using the QIIME2 software package [16]. Raw sequence data were filtered, de-replicated, and denoised to generate ASV tables using DADA2 implemented in QIIME2 [17]. NCBI RefSeq (version 2019.2.1) was used as the taxonomic database. Downstream analysis was conducted using the QIIME2 and R packages [18, 19]. The alpha diversity of the gut bacteria was evaluated using three indices: Shannon’s index, Pielou’s evenness, and effective number of species (ENS)/probability of interspecific encounter (PIE). The beta diversity of the canine gut microbiota was analyzed using Bray–Curtis dissimilarity. Differential abundance analysis of the gut microbiota was performed using linear discriminant analysis effect size with a cut-off value of *p* < 0.05 and |LDA|> 2 [20]. Correlation analysis between the clinical scores of cAD and gut microbiota was performed using the Spearman rank correlation method. To predict the abundance of gene families and higher-level pathways present in microbial communities, phylogenetic investigation of communities by reconstruction of unobserved state 2 (PICRUSt2) was performed based on the MetaCyc ontology database [21]. Hidden state prediction was performed using the maximum parsimony method, and the maximum nearest sequenced taxon index was set to two to remove sequences with a value above the cut-off.

Categorical variables (sex) of participant dogs were compared using a chi-square test, while continuous variables (age and body weight) were analyzed using a *t*-test. The Friedman Test was used for longitudinal comparisons of alpha diversity and clinical scores. Permutational multivariate analysis of variance (PERMANOVA) was used to analyze differences in beta diversity. Statistical significance was set at *p* < 0.05. Statistical analysis was performed using R version 3.6.3 and GraphPad Prism version 10 (CA, USA).

## Results

### General characteristics of participant dogs

There were no significant differences in potential confounding factors, including age, body weight, and sex, between the healthy and cAD groups (Table 1). Furthermore, principal-coordinate analysis (PCoA) using Bray–Curtis dissimilarity revealed no significant differences in gut microbiota based on these factors (*p* > 0.05, PERMANOVA; Table S2). Additionally, there was no significant side effects or clinical symptoms during probiotic administration reported by owner.

**Table 1:** Demographics of participant dogs

Variable	cAD (*n* = 23)	Healthy (*n* = 11)	*P-*value
Body weight (kg) ^a^	4.80 ± 2.24	6.60 ± 2.41	> 0.05
Age (years) ^b^	4.83 ± 3.58	2.98 ± 2.40	> 0.05
Sex (*n*)			> 0.05 ^c^
Male	11	7	
Female	12	4	

^a^Body weight value is the mean ± standard deviation; *p*-value was evaluated using a Mann–Whitney U test

^b^Age value is the median ± standard deviation; *p*-value was evaluated using a t-test

^c^*P*-value was evaluated using a Chi-square test

### Gut microbiota dysbiosis in dogs with cAD

**Fig. 2:**
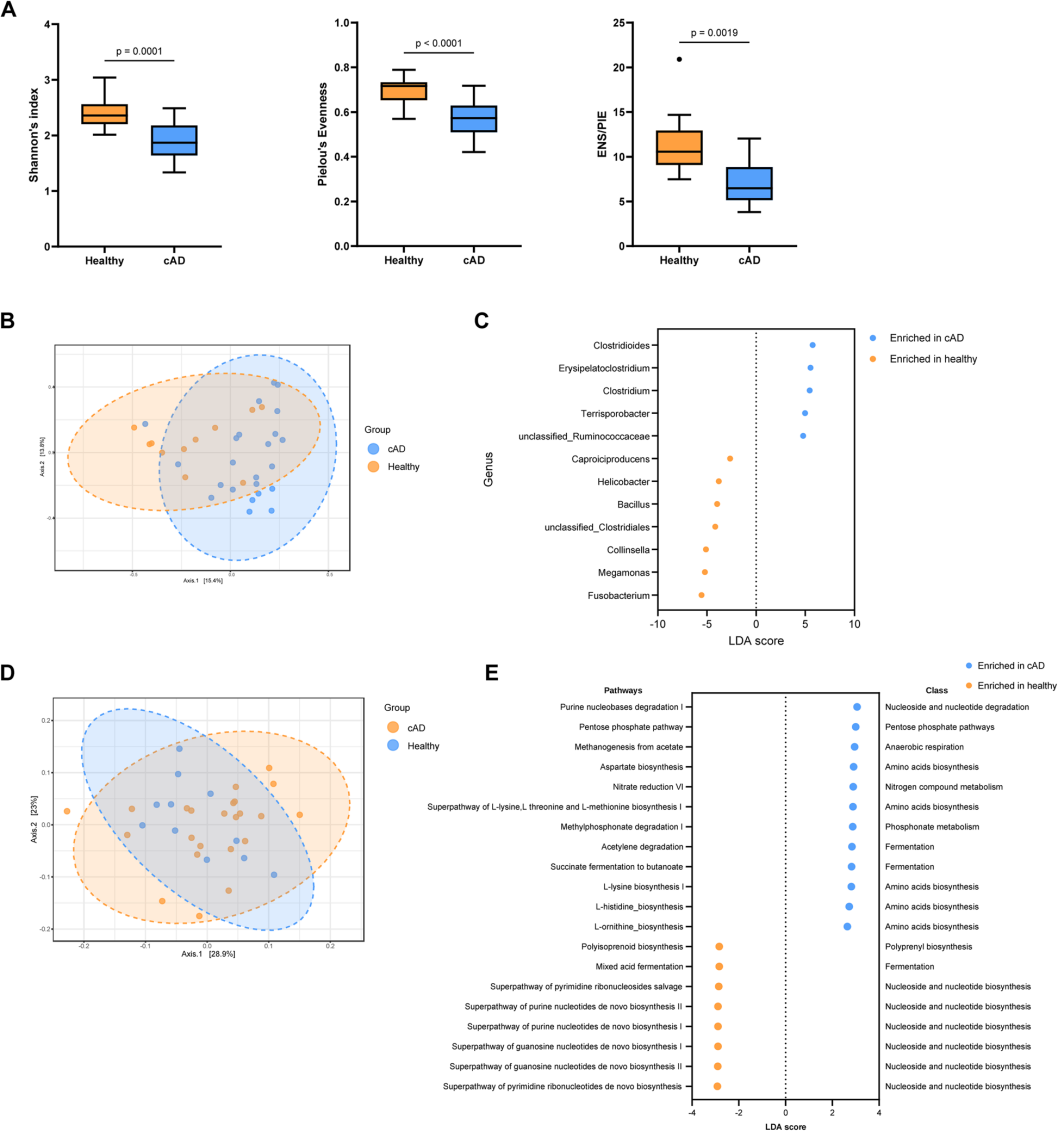
Comparative analysis of the gut microbiota between dogs with cAD and healthy dogs. **A** Box plots demonstrating the gut microbiota alpha diversity of dogs with cAD and healthy dogs. **B** Principal coordinate analysis (PCoA) plot based on Bray–Curtis dissimilarity of the gut microbiota of dogs with cAD and healthy dogs prior to probiotic administration. **C** Differential abundance analysis of the gut microbiota at genus level using linear discriminant analysis effect size. Genera with LDA scores > 2 and *p* < 0.05 are shown. **D** PCoA plot based on Bray–Curtis dissimilarity of the metabolic pathway of the gut microbiota in dogs with cAD and healthy dogs prior to probiotic administration. **E** Differential abundance analysis of the metabolic pathways of the gut microbiota using LDA effect size. Top 20 pathways with significant difference (|LDA) > 2, and *p* < 0.05) are shown

To investigate gut microbiota dysbiosis in cAD, we compared the gut microbiota of healthy dogs with those with cAD at baseline. Alpha diversity analysis revealed that Shannon’s index, Pielou’s evenness index, and the ENS/PIE metric were significantly lower (*p* < 0.05) in dogs with cAD than in healthy dogs (Fig. 2A). In addition, beta diversity analysis based on Bray–Curtis dissimilarity revealed a significant difference (*p* < 0.05) between the gut microbiota of healthy dogs and those with cAD (Fig. 2B). Differential abundance analysis showed that the abundance of 12 genera was significantly different (*p* < 0.05, |LDA| score > 2) between healthy dogs and dogs with cAD. Seven genera, including *Fusobacterium*, *Megamonas*, *Collinsella*, *unclassified Clostridiales*, *Bacillus*, *Helicobacter,* and *Caproiciproducens,* were significantly more abundant in healthy dogs. In contrast, *Clostridioides*, *Erysipelatoclostridium, Clostridium, Terrisporobacter,* and *unclassified Ruminococcaceae* were significantly more abundant in dogs with cAD (Fig. 2C).

We further compared the metabolic pathways of the gut microbiota of healthy dogs and those with cAD at baseline. Beta diversity of the metabolic pathways of the gut microbiota based on Bray–Curtis dissimilarity revealed no significant difference (*p* > 0.05) between healthy dogs and those with cAD (Fig. 2D). In contrast, differential abundance analysis showed that the abundance of 46 metabolic pathways were significantly different (*p* < 0.05, |LDA| score > 2) between healthy dogs and dogs with cAD (Fig. 2E). In cAD dogs, 27 metabolic pathways, including anhydromuropeptide recycling, purine nucleobase degradation, methanogenesis, aspartate biosynthesis and nitrate reduction, and the superpathways of amino acid biosynthesis were significantly enriched compared to those in healthy dogs. In healthy dogs, 19 metabolic pathways were significantly enriched compared to those in cAD dogs. Most enriched metabolic pathways were involved in de novo biosynthesis of pyrimidine ribonucleotides, guanosine nucleotides, and purine nucleotides.

### Gut microbiota dysbiosis is associated with cAD clinical parameters

**Fig. 3:**
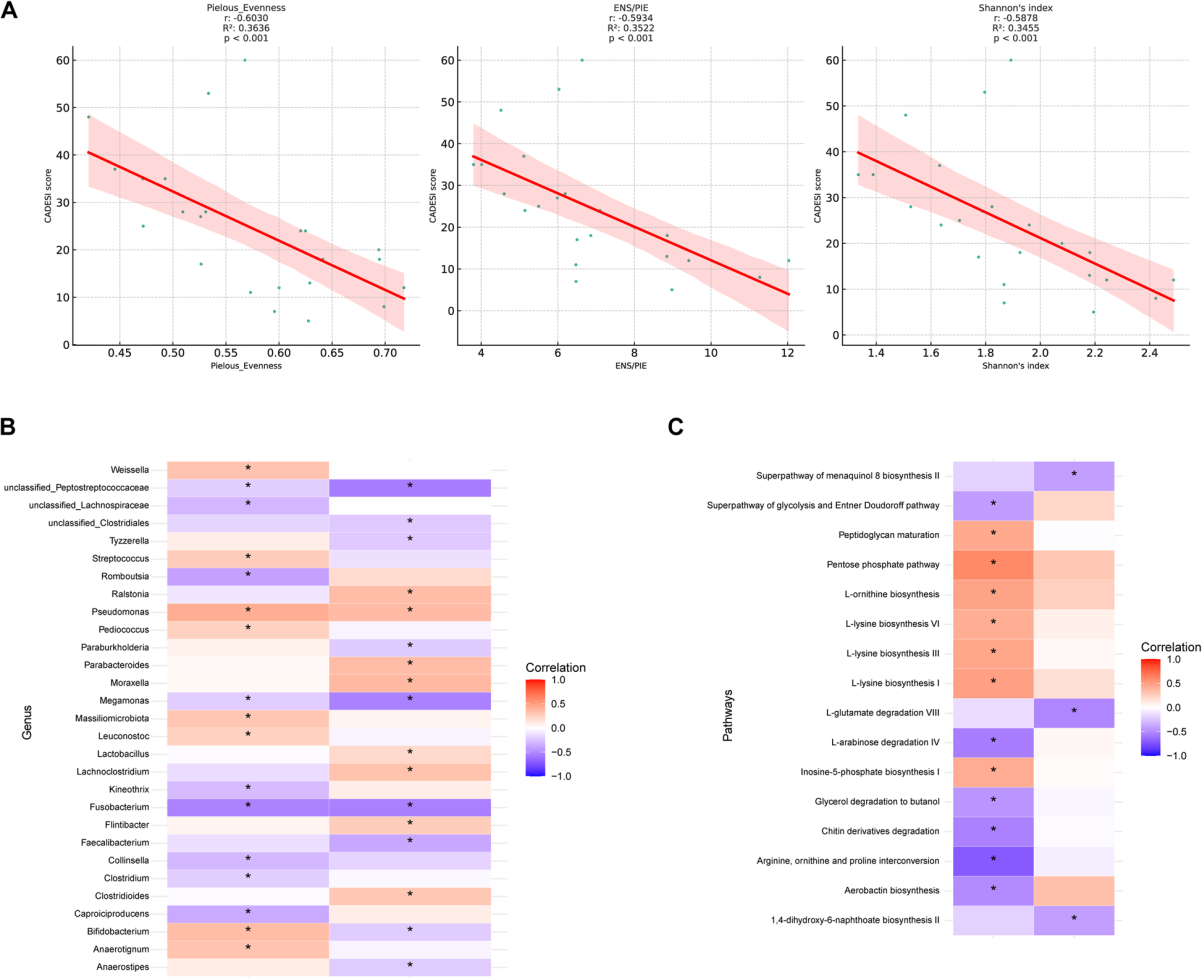
Correlation analysis of the gut microbiota and cAD clinical scores. **A** Spearman rank correlation plot of alpha diversity indices and Canine Atopic Dermatitis Extent and Severity Index (CADESI-4) score. Heatmap illustrating correlation of clinical scores (CADESI- 04 and PVAS), **B** gut microbial taxa, and **C** metabolic pathway. Significant correlations (*p* < 0.05) are denoted with asterisks

To investigate the association between gut microbiota dysbiosis and cAD, we performed a correlation analysis between the alpha diversity and the CADESI scores of dogs with cAD. The CADESI-04 score showed a significant negative correlation (*p* < 0.05) with all three alpha diversity indices (Fig. 3A). The PVAS score showed no significant correlation (*p* > 0.05) with any of the three alpha diversity indices.

We performed a further correlation analysis between gut microbe abundance, metabolic pathways, and the CADESI and PVAS scores of dogs with cAD to identify marker microbes associated with cAD severity. A total of 29 genera showed a significant correlation (*p* < 0.05) with CADESI or PVAS scores (Fig. 3B). Eight genera, including *Weissella*, *Streptococcus*, *Pediococcus*, *Leuconostoc*, *Fusobacterium*, *Megamonas,* and unclassified *Peptostreptococcaceae* showed negative correlations with both CADESI and PVAS scores, whereas *Pseudomonas* showed positive correlations with both indices. Moreover, 16 metabolic pathways showed a significant correlation (*p* < 0.05) with CADESI or PVAS scores (Fig. 3C). The abundance of seven metabolic pathways, including peptidoglycan maturation, pentose phosphate pathway, L-ornithine biosynthesis, L-lysine biosynthesis VI, L-lysine biosynthesis III, L-lysine biosynthesis I, and inosine-5-phosphate biosynthesis I, positively correlated with the CADESI score. The abundance of seven metabolic pathways, including the glycolysis superpathway, the Entner Doudoroff pathway, L-arabinose degradation, glycerol degradation, chitin derivatives degradation, arginine, ornithine, and proline interconversion, and aerobactin biosynthesis negatively correlated with the CADESI score. In addition, three metabolic pathways, including the superpathway of menaquinol 8 biosynthesis II, L-glutamate degradation, and 1,4-dihydroxy-6-naphthoate biosynthesis, negatively correlated with PVAS.

### Probiotic administration ameliorates the clinical symptoms of cAD

**Fig. 4:**
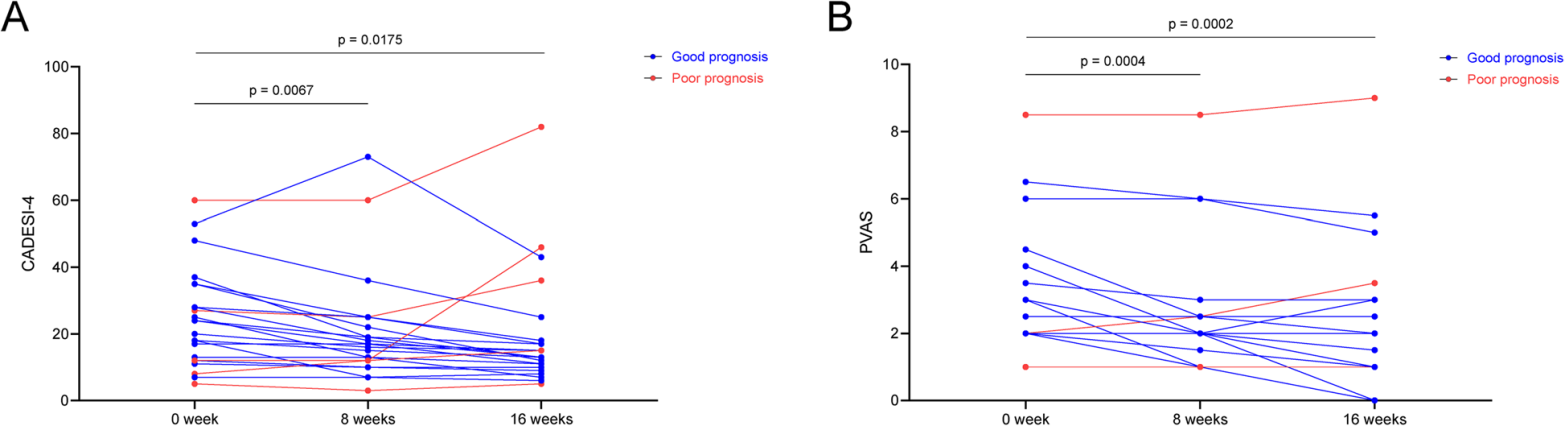
Clinical score alteration during probiotic administration. Aligned dot plot demonstrating the alteration of **A** Canine Atopic Dermatitis Extent and Severity Index score, **B** Pruritis Visual Analog Scale during probiotic administration. Wilcoxon’s signed-rank test was performed to compare clinical scores

The CADESI scores of dogs with cAD at baseline varied from 3 to 60, with a mean and standard deviation of 24.57 ± 14.74. After 8 weeks of probiotic administration, CADESI scores significantly decreased (*p* < 0.05) to 20.61 ± 16.31 when compared with that of baseline. After 16 weeks of administration, CADESI scores significantly decreased (*p* < 0.05) to 19.04 ± 17.06 (Fig. 4A). The CADESI scores of seven dogs were reduced by more than 50% 16 weeks following probiotic administration. The PVAS score varied from 0 to 6.5 and was 2.78 ± 1.48 in baseline but decreased significantly to 2.17 ± 1.39 and 1.80 ± 1.40 after 8 and 16 weeks of probiotic administration, respectively (Fig. 4B).

### Gut microbiota alteration during probiotic administration

**Fig. 5:**
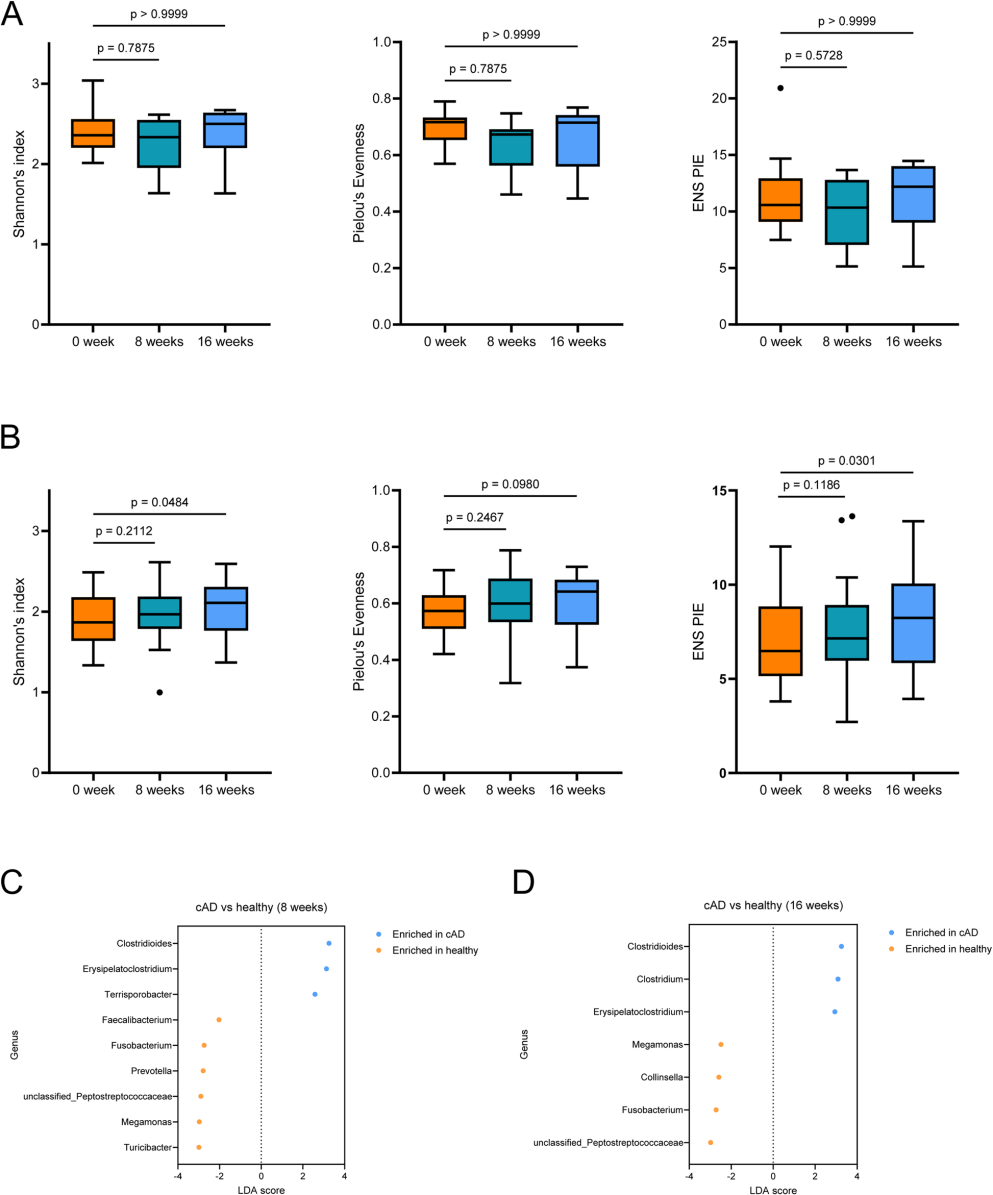
Gut microbiota alteration during probiotic administration. Alteration of alpha diversity indices, including Shannon’s index, Pielou’s Evenness, and ENS/PIE of the gut microbiota in **A** healthy dogs and **B** those with cAD. Differential abundance analysis of the gut microbial genera using linear discriminant analysis effect size between healthy dogs and dogs with cAD **C** 8 weeks and **D** 16 weeks after probiotic administration. ENS, effective number of species; PIE, probability of interspecific encounter; cAD, canine atopic dermatitis

In healthy dogs, all alpha diversity indices showed no significant differences (*p* > 0.05) compared to those at baseline at both 8 and 16 weeks (Fig. 5A). In dogs with cAD, alpha diversity did not significantly increase (*p* > 0.05) compared to that at baseline. In contrast, Shannon’s index and ENS/PIE significantly increased (*p* < 0.05) 16 weeks after probiotic administration in healthy dogs and dogs with cAD (Fig. 5B). All alpha diversity indices of the gut microbiota of dogs with cAD were significantly lower (*p* < 0.05) than those of healthy dogs.

Differential abundance analysis showed that the abundance of nine genera was significantly different (*p* < 0.05, |LDA score|> 2) between healthy dogs and dogs with cAD after 8 weeks of probiotic administration (Fig. 5C). Seven genera differed significantly (*p* < 0.05, |LDA score|> 2) after 16 weeks of probiotic administration (Fig. 5D). No genera showed significant differences between time points when the gut microbiotas of dogs with cAD were compared.

### Alteration patterns of the gut microbiota are associated with prognosis

**Fig. 6:**
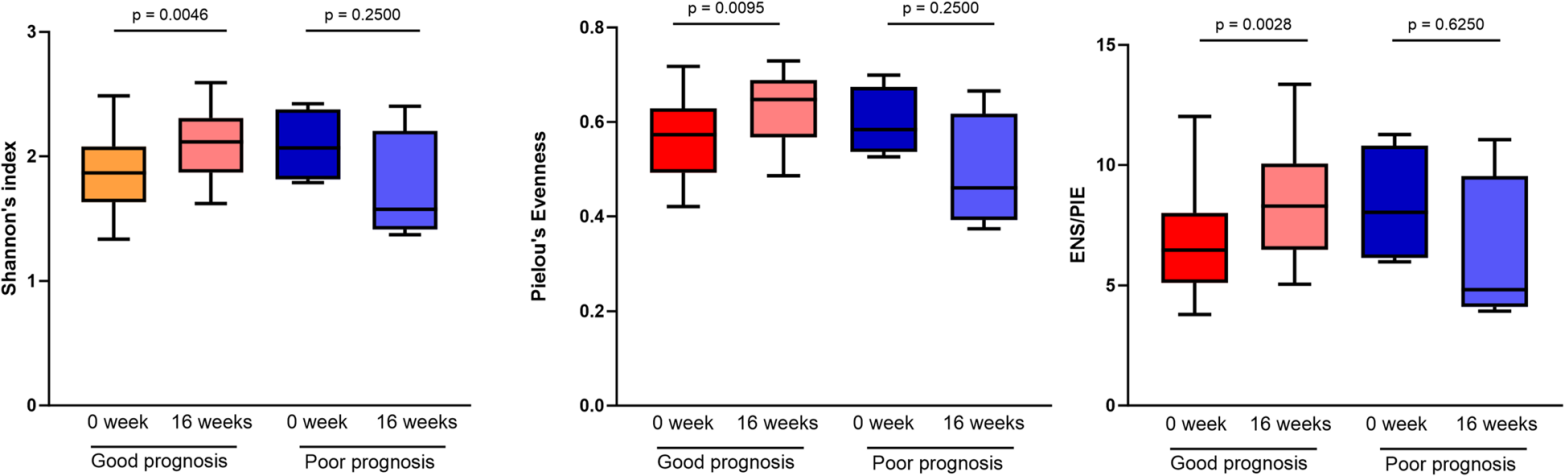
Alteration of gut microbiota alpha diversity according to the probiotic administration prognosis. Three indices of the alpha diversity, including Shannon’s index, Pielou’s Evenness, and ENS/PIE 0 and 16 weeks after probiotic administration, are shown in the box plot. ENS, effective number of species; PIE, probability of interspecific encounter

According to the CADES-4 analysis, the clinical symptoms of 19 of the 23 dogs that suffered from cAD improved, whereas those of four dogs showed no effect after 16 weeks of probiotic administration. We hypothesized that the altered pattern of the gut microbiota is associated with differences in prognosis. To test this hypothesis, dogs with cAD were divided into two groups: 1) dogs with improved symptoms (good prognosis group) and 2) dogs with no change or worsening symptoms (poor prognosis group). All alpha diversity indices significantly increased (*p* < 0.05) in the good prognosis group, whereas no significant differences were observed in all alpha diversity indices in the poor prognosis group (Fig. 6).

## Discussion

While established immune-suppressing therapies exist for the treatment of cAD, they have side effects, such as infections [22, 23]. The disease severely impairs the quality of life of dogs. Consequently, novel management strategies are required to complement existing treatments. Recent advances in microbiome research have revealed that alterations in the gut microbiota can trigger various chronic skin diseases through the gut–skin axis [24, 25]. Previous studies in dogs with cAD have highlighted changes in the gut and skin microbiota [2, 3], suggesting that modulating the microbiota in dogs may be a viable approach for managing cAD. Therefore, this study aimed to evaluate probiotic administration as a treatment option for cAD by investigating its effects on the clinical symptoms and gut microbiota of dogs with cAD. To the best of our knowledge, this is the first study to reveal alterations in the gut microbiota of dogs with cAD following probiotic administration.

The results at baseline indicated that dogs with cAD possessed a distinct gut microbiota compared with that od healthy dogs, including a significant decrease in the alpha diversity of their gut microbiota. This decrease may be because the commensal bacteria that maintain intestinal homeostasis may have reduced. Although the definition of dysbiosis in microbiota is controversial, a decrease in alpha diversity is a consistent finding across most studies [26, 27]. Thus, our findings demonstrated gut microbiota dysbiosis in dogs with cAD by showing low microbiota diversity. Moreover, the enrichment or depletion of specific bacteria is an indicator of gut microbiota dysbiosis [28]. Differential abundance analysis revealed that *Fusobacterium* and *Megamonas* were significantly depleted in dogs with cAD. This finding is consistent with that of a previous study on the gut microbiota of dogs with cAD [3].

*Megamonas* is known to produce short-chain fatty acids, including acetate and propionate, through fermentation, which positively affects host health [29]. Consistently, a higher level of propionate in feces may be associated with a reduced risk of atopy in early life in humans [30]. Moreover, our results showed that *Collinsella* and *Helicobacter* were significantly depleted in the gut microbiota of cAD dogs. Notably, the abundance of *Collinsella* is negatively associated with the severity of atopic dermatitis in human infants [31]. Furthermore, a previous study using a mouse model of atopic dermatitis showed that *Helicobacter* infection protects against atopic dermatitis by regulating the immune response in the gastrointestinal tract [32]. Therefore, the depletion of *Fusobacterium*, *Megamonas, Collinsella,* and *Helicobacter* may play a role in cAD pathogenesis. In contrast, our results showed that *Clostridioides* and *Terrisporobacter* were enriched in the gut microbiota of cAD dogs. This result is consistent with previous reports of the increased abundance of *Clostridioides* and *Terrisporobacter* in the gut microbiota of patients with AD [33, 34], demonstrating a possible role of these microbes in cAD pathogenesis.

Our results also showed that the metabolic pathways involving the gut microbiota in cAD dogs were distinct from those in healthy dogs. Metabolic pathways involved in the biosynthesis of amino acids, including L-histidine, were significantly enriched in cAD dogs, consistent with previous studies on human AD and AD mouse models [35, 36]. Histidine biosynthesis requires phosphoribosylpyrophosphate (PRPP), a crucial precursor for nucleic acids, proteins, and NAD(P) coenzymes. The expression of PRPP synthetase in patients with AD is higher than in healthy controls [37], suggesting that PRPP formation may play a role in the metabolic regulation of AD. This upregulation in PRPP biosynthesis could reflect the altered metabolic demands associated with the condition, further supporting the possibility that amino acid metabolism is closely linked to the pathophysiology of cAD.

Additionally, our results showed that dogs with cAD not only exhibited gut microbiome dysbiosis but also demonstrated a significant negative correlation with microbial diversity as the symptoms worsened. The degree of dysbiosis and clinical scores were also significantly correlated. This suggests that the gut microbiota may act as a potential cause of cAD and contribute to the exacerbation of symptoms. Microbes that were negatively correlated with disease severity were *Fusobacterium* and *Megamonas*, genera that were depleted in dogs with cAD. Therefore, the depletion of these microbes may play a major role in cAD pathogenesis. In addition, we found a positive correlation between *Lactobacillus* and *Bifidobacterium* abundances and CADESI or PVAS, indicating that higher abundances of these typically beneficial microbes were associated with more severe atopic dermatitis. While *Lactobacillus* and *Bifidobacterium* are linked to health-promoting effects [38], their increased presence in dogs with more severe disease may reflect a compensatory response to inflammation or immune dysregulation. Similar observations have been reported in human patients with AD, where the abundance of beneficial short-chain fatty acid producers, including *Bacteroides* and *Parabacteroides* [37, 38] positively correlates with AD severity [31].

In the present study, 16 weeks of probiotic administration significantly improved clinical scores in dogs with cAD and increased the gut microbiota alpha diversity in dogs with cAD, whereas the gut microbiota alpha diversity in healthy dogs showed no changes. The gut microbiota of healthy dogs maintains homeostasis; however, in dogs with cAD, homeostasis is disrupted because of dysbiosis. Additionally, previous studies have demonstrated that increased alpha diversity is associated with disease amelioration [39]. Therefore, the increased gut microbiota diversity was owing to dysbiosis modulation by probiotics. Although no significant differences were observed in healthy dogs after 16 weeks of administering probiotics, the detected significant increase in dogs with cAD suggests that long-term probiotic administration may not show significant differences when compared with healthy dogs. Further studies on long-term probiotic administration may yield interesting results. Alteration pattern analysis found that dogs with improved prognoses showed a significant increase in gut microbiota diversity, whereas those with no improvement did not show an increase in diversity. These findings suggest that when probiotics improve gut microbiota dysbiosis, atopy is ameliorated; however, when the gut microbiota shows no changes, atopy is not ameliorated.

This study focused on household dogs that lived with their owners, as opposed to laboratory dogs raised in controlled environments. Owing to consistent external and host factors, the gut microbiota of laboratory dogs show less variation among individuals. Laboratory dogs exhibit less diversity in factors such as sex, age, weight, and physical health, which may limit the generalization of results. This study enhanced result reproducibility by focusing on household dogs, reflecting a broader spectrum of dog populations. Therefore, this study provides a dependable overview of dogs and offers insights into the influence of probiotics on gut microbiota changes and their benefits in dogs with cAD.

This study did not include dogs with cAD that had not undergone probiotic administration, which may be a limitation. As all study subjects were privately owned dogs that visited veterinary hospitals, establishing a cAD group that did not receive any treatment was difficult. Comparative analysis of the clinical score and gut microbiome between dogs with cAD that were administered probiotics and those that were not may provide a more comprehensive understanding of the role of gut microbiome alterations due to probiotics in cAD. Additionally, as individual gut microbiota is associated with drug susceptibility for disease treatment [40], studying the combined effects of drugs such as oclacitinib and probiotics may be necessary. Finally, future research employing shotgun sequencing for microbiome function analysis and multi-omics analysis for microbial metabolite analysis could provide further information regarding host-microbe interactions in dogs with cAD.

## Conclusions

This study demonstrated an association between gut microbiota dysbiosis and cAD. Disease severity is significantly correlated with the diversity and abundance of marker microbes, which supports this association. Furthermore, probiotic administration in dogs with cAD significantly improved clinical symptoms via increasing alpha diversity. In conclusion, the present study shows that probiotic administration ameliorates cAD by modulating gut microbiota. This study provides insights into novel microbiota-targeted management strategies for cAD treatment.

## Supplementary Information

Additional file 1. Contains supplementary tables.

Additional file 2. Contains Supplementary figure.

## Data Availability

The datasets generated and/or analyzed during the current study are available in the SRA repository under the Project Accession ID of PRJNA810286.

## References

[1] Bizikova P, Santoro D, Marsella R, Nuttall T, Eisenschenk MNC, Pucheu-Haston CM. Review: clinical and histological manifestations of canine atopic dermatitis. Vet Dermatol. 2015;26:79-e24. 25676252 10.1111/vde.12196

[2] Rostaher A, Morsy Y, Favrot C, Unterer S, Schnyder M, Scharl M, et al. Comparison of the gut microbiome between atopic and healthy dogs—preliminary data. Animals. 2022;12:2377. 36139237 10.3390/ani12182377 PMC9495170

[3] Thomsen M, Künstner A, Wohlers I, Olbrich M, Lenfers T, Osumi T, et al. A comprehensive analysis of gut and skin microbiota in canine atopic dermatitis in Shiba Inu dogs. Microbiome. 2023;11:232. 37864204 10.1186/s40168-023-01671-2 PMC10590023

[4] Uchiyama J, Osumi T, Mizukami K, Fukuyama T, Shima A, Unno A, et al. Characterization of the oral and faecal microbiota associated with atopic dermatitis in dogs selected from a purebred Shiba Inu colony. Lett Appl Microbiol. 2022;75:1607–16. 36067033 10.1111/lam.13828

[5] O’Toole PW, Marchesi JR, Hill C. Next-generation probiotics: the spectrum from probiotics to live biotherapeutics. Nat Microbiol. 2017;2:1–6. 10.1038/nmicrobiol.2017.57 28440276

[6] Mathipa MG, Thantsha MS. Probiotic engineering: towards development of robust probiotic strains with enhanced functional properties and for targeted control of enteric pathogens. Gut Pathog. 2017;9:1–17. 28491143 10.1186/s13099-017-0178-9 PMC5422995

[7] Ma T, Shen X, Shi X, Sakandar HA, Quan K, Li Y, et al. Targeting gut microbiota and metabolism as the major probiotic mechanism-an evidence-based review. Trends Food Sci Technol. 2023;138:178–98.

[8] Bae H, Hwang TS, Lee HC, Jung DI, Kim SH, Yu D. Successful treatment of canine infective endocarditis caused by Bacillus amyloliquefaciens. Vet Q. 2022;42:41–7. 35068361 10.1080/01652176.2022.2033879 PMC8843097

[9] Jugan MC, Rudinsky AJ, Parker VJ, Gilor C. Use of probiotics in small animal veterinary medicine. J Am Vet Med Assoc. 2017;250:519–28. 28207322 10.2460/javma.250.5.519

[10] Xu H, Huang W, Hou Q, Kwok LY, Laga W, Wang Y, et al. Oral administration of compound probiotics improved canine feed intake, weight gain, immunity and intestinal microbiota. Front Immunol. 2019;10:394673. 10.3389/fimmu.2019.00666 PMC6454072 31001271

[11] Kang A, Kwak MJ, Lee DJ, Lee JJ, Kim MK, Song M, et al. Dietary supplementation with probiotics promotes weight loss by reshaping the gut microbiome and energy metabolism in obese dogs. Microbiol Spectr. 2024;12:e02552-e2623. 38270436 10.1128/spectrum.02552-23 PMC10913549

[12] Marsella R, Santoro D, Ahrens K. Early exposure to probiotics in a canine model of atopic dermatitis has long-term clinical and immunological effects. Vet Immunol Immunopathol. 2012;146:185–9. 22436376 10.1016/j.vetimm.2012.02.013

[13] Kim H, Rather IA, Kim H, Kim S, Kim T, Jang J, et al. A double-blind, placebo controlled-trial of a probiotic strain Lactobacillus sakei probio-65 for the prevention of canine atopic dermatitis. J Microbiol Biotechnol. 2015;25:1966–9. 26282691 10.4014/jmb.1506.06065

[14] Olivry T, Saridomichelakis M, Nuttall T, Bensignor E, Griffin CE, Hill PB, et al. Validation of the Canine Atopic Dermatitis Extent and Severity Index (CADESI)-4, a simplified severity scale for assessing skin lesions of atopic dermatitis in dogs. Vet Dermatol. 2014;25:77-e25. 24461108 10.1111/vde.12107

[15] Song H, Lee J, Yi S, Kim WH, Kim Y, Namgoong B, et al. Red ginseng dietary fiber shows prebiotic potential by modulating gut microbiota in dogs. Microbiol Spectr. 2023;11:e00949-e1023. 37367492 10.1128/spectrum.00949-23 PMC10433987

[16] Bolyen E, Rideout JR, Dillon MR, Bokulich NA, Abnet CC, Al-Ghalith GA, et al. Reproducible, interactive, scalable and extensible microbiome data science using QIIME 2. Nat Biotechnol. 2019;37:852–7. 31341288 10.1038/s41587-019-0209-9 PMC7015180

[17] Callahan BJ, McMurdie PJ, Rosen MJ, Han AW, Johnson AJA, Holmes SP. DADA2: high-resolution sample inference from Illumina amplicon data. Nat Methods. 2016;13:581–3. 27214047 10.1038/nmeth.3869 PMC4927377

[18] Chong J, Liu P, Zhou G, Xia J. Using MicrobiomeAnalyst for comprehensive statistical, functional, and meta-analysis of microbiome data. Nat Protoc. 2020;15:799–821. 31942082 10.1038/s41596-019-0264-1

[19] McMurdie PJ, Holmes S. Phyloseq: an R package for reproducible interactive analysis and graphics of microbiome census data. PLoS ONE. 2013;8:e61217. 23630581 10.1371/journal.pone.0061217 PMC3632530

[20] Segata N, Izard J, Waldron L, Gevers D, Miropolsky L, Garrett WS, et al. Metagenomic biomarker discovery and explanation. Genome Biol. 2011;12:R60. 21702898 10.1186/gb-2011-12-6-r60 PMC3218848

[21] Douglas GM, Maffei VJ, Zaneveld JR, Yurgel SN, Brown JR, Taylor CM, et al. PICRUSt2 for prediction of metagenome functions. Nat Biotechnol. 2020;38:685–8. 32483366 10.1038/s41587-020-0548-6 PMC7365738

[22] Linek M, Favrot C. Impact of canine atopic dermatitis on the health-related quality of life of affected dogs and quality of life of their owners. Vet Dermatol. 2010;21:456–62. 20492625 10.1111/j.1365-3164.2010.00899.x

[23] Saridomichelakis MN, Olivry T. An update on the treatment of canine atopic dermatitis. Vet J. 2016;207:29–37. 26586215 10.1016/j.tvjl.2015.09.016

[24] Mahmud MR, Akter S, Tamanna SK, Mazumder L, Esti IZ, Banerjee S, et al. Impact of gut microbiome on skin health: gut-skin axis observed through the lenses of therapeutics and skin diseases. Gut Microbes. 2022;14:2096995. 35866234 10.1080/19490976.2022.2096995 PMC9311318

[25] Salem I, Ramser A, Isham N, Ghannoum MA. The gut microbiome as a major regulator of the gut-skin axis. Front Microbiol. 2018;9:1459. 30042740 10.3389/fmicb.2018.01459 PMC6048199

[26] Hooks KB, O’Malley MA. Dysbiosis and its discontents. MBio. 2017;8:10–1128. 10.1128/mBio.01492-17 PMC5635691 29018121

[27] Reese AT, Dunn RR. Drivers of microbiome biodiversity: a review of general rules, feces, and ignorance. MBio. 2018;9:e01294-e1318. 30065092 10.1128/mBio.01294-18 PMC6069118

[28] Litvak Y, Byndloss MX, Tsolis RM, Bäumler AJ. Dysbiotic Proteobacteria expansion: a microbial signature of epithelial dysfunction. Curr Opin Microbiol. 2017;39:1–6. 28783509 10.1016/j.mib.2017.07.003

[29] Yang X, Zhang M, Liu Y, Wei F, Li X, Feng Y, et al. Inulin-enriched Megamonas funiformis ameliorates metabolic dysfunction-associated fatty liver disease by producing propionic acid. NPJ Biofilms Microbiomes. 2023;9:84. 37925493 10.1038/s41522-023-00451-y PMC10625582

[30] Roduit C, Frei R, Ferstl R, Loeliger S, Westermann P, Rhyner C, et al. High levels of butyrate and propionate in early life are associated with protection against atopy. Allergy. 2019;74:799–809. 30390309 10.1111/all.13660

[31] Liu X, Cai M, Chen M, Chen J, Zhu T, Wu S, et al. Alterations in gut microbiome associated with severity of atopic dermatitis in infants. Australas J Dermatol. 2024;65:328–36. 38419203 10.1111/ajd.14237

[32] Xue Q, Li X, Li Y, Xu J, Wu Z, Wang J. Dialogue between gastrointestinal tract and skin: new insights into the Helicobacter pylori and atopic dermatitis. Helicobacter. 2021;26:e12771. 33368906 10.1111/hel.12771

[33] Sasaki M, Schwab C, Ramirez Garcia A, Li Q, Ferstl R, Bersuch E, et al. The abundance of Ruminococcus bromii is associated with faecal butyrate levels and atopic dermatitis in infancy. Allergy. 2022;77:3629–40. 35917214 10.1111/all.15440 PMC10087690

[34] Thirion F, Guilly S, Fromentin S, Plaza Oñate F, Alvarez AS, Le Chatelier E, et al. Changes in gut microbiota of patients with atopic dermatitis during balneotherapy. Clin Cosmet Investig Dermatol. 2022;15:163–76. 35140493 10.2147/CCID.S342104 PMC8818552

[35] Kim W, Jang YJ, Park S, Min S, Kwon H, Jo MJ, et al. Lactobacillus acidophilus KBL409 ameliorates atopic dermatitis in a mouse model. J Microbiol. 2024;62:91–9. 38386273 10.1007/s12275-024-00104-5 PMC11021314

[36] Patumcharoenpol P, Kingkaw A, Nakphaichit M, Chatchatee P, Suratannon N, Panagiotou G, et al. Exploring longitudinal gut microbiome towards metabolic functional changes associated in atopic dermatitis in early childhood. Biology (Basel). 2023;12:1262. 37759661 10.3390/biology12091262 PMC10525566

[37] Kingkaw A, Nakphaichit M, Suratannon N, Nitisinprasert S, Wongoutong C, Chatchatee P, et al. Analysis of the infant gut microbiome reveals metabolic functional roles associated with healthy infants and infants with atopic dermatitis using metaproteomics. PeerJ. 2020;8:e9988. 33033661 10.7717/peerj.9988 PMC7521340

[38] Messaoudi M, Violle N, Bisson JF, Desor D, Javelot H, Rougeot C. Beneficial psychological effects of a probiotic formulation (Lactobacillus helveticus R0052 and Bifidobacterium longum R0175) in healthy human volunteers. Gut Microbes. 2011;2:256–61. 21983070 10.4161/gmic.2.4.16108

[39] Ma Z, Li L, Gotelli NJ. Diversity-disease relationships and shared species analyses for human microbiome-associated diseases. ISME J. 2019;13:1911–9. 30894688 10.1038/s41396-019-0395-y PMC6775969

[40] Wilson ID, Nicholson JK. Gut microbiome interactions with drug metabolism, efficacy, and toxicity. Transl Res. 2017;179:204–22. 27591027 10.1016/j.trsl.2016.08.002 PMC5718288

