# Supplementary material for: Probiotics ameliorate atopic dermatitis by modulating the dysbiosis of the gut microbiota in dogs
Source: BMC Microbiol. 2025 Apr 22;25:228. doi: 10.1186/s12866-025-03924-6 (PMC12012994; doi:10.1186/s12866-025-03924-6)
Supplement: Supplementary file 2 — Additional file 2. Contains Supplementary figure. [file 12866_2025_3924_MOESM2_ESM.docx]

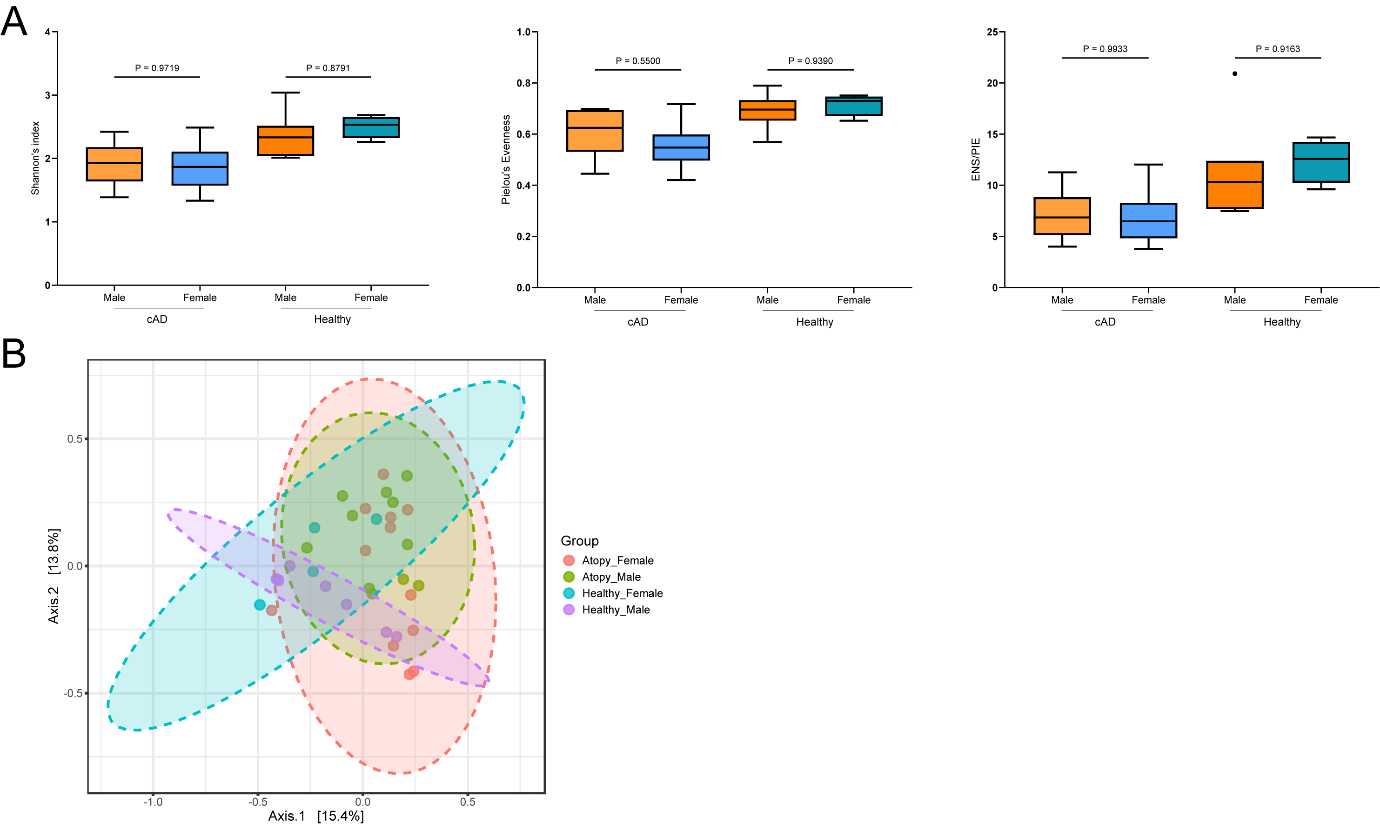


**Figure S1. Comparative analysis of the gut microbiota of healthy and cAD dogs at baseline. (A) Box** (A) Box plots demonstrating the gut microbiota alpha diversity of dogs with cAD and healthy dogs according to sex. (B) Principal coordinate analysis (PCoA) plot based on Bray–Curtis dissimilarity of the gut microbiota of dogs with cAD and healthy dogs at baseline.
